# The Presentation Location of the Reference Stimuli Affects the Left-Side Bias in the Processing of Faces and Chinese Characters

**DOI:** 10.3389/fpsyg.2017.01673

**Published:** 2017-09-26

**Authors:** Chenglin Li, Xiaohua Cao

**Affiliations:** Department of Psychology, Zhejiang Normal University, Jinhua, China

**Keywords:** left-side bias, visual field, face, Chinese character, perception

## Abstract

For faces and Chinese characters, a left-side processing bias, in which observers rely more heavily on information conveyed by the left side of stimuli than the right side of stimuli, has been frequently reported in previous studies. However, it remains unclear whether this left-side bias effect is modulated by the reference stimuli's location. The present study adopted the chimeric stimuli task to investigate the influence of the presentation location of the reference stimuli on the left-side bias in face and Chinese character processing. The results demonstrated that when a reference face was presented in the left visual field of its chimeric images, which are centrally presented, the participants showed a preference higher than the no-bias threshold for the left chimeric face; this effect, however, was not observed in the right visual field. This finding indicates that the left-side bias effect in face processing is stronger when the reference face is in the left visual field. In contrast, the left-side bias was observed in Chinese character processing when the reference Chinese character was presented in either the left or right visual field. Together, these findings suggest that although faces and Chinese characters both have a left-side processing bias, the underlying neural mechanisms of this left-side bias might be different.

## Introduction

Adults are experts at recognizing human faces and words. Many behavioral effects have been used to investigate the mechanism of the perceptual expertise, for instance, the inversion effect (e.g., Yin, 1969; Haxby et al., 1999), the composite effect (for a review, see Richler and Gauthier, 2014), and the left-side bias (e.g., Gilbert and Bakan, 1973; Hsiao and Cottrell, 2009; Proietti et al., 2015). The left-side bias refers to the process by which a chimeric face created from the left side of a face (from the viewer's perspective) and its mirror image are considered more similar to the original/reference face than a chimeric face created from the right side of the same face and its mirror image. In a facial expression judgment task, Wolff (1933) first observed the left-side bias effect. This effect was later replicated by Gilbert and Bakan (1973) in a face perception judgment task, and it has been found in tasks involving the processing of other aspects of faces, such as facial identity (e.g., Coolican, et al., 2008; Proietti et al., 2015), emotion (e.g., David, 1993; Ferber and Murray, 2005; Bourne, 2008, 2011; Coolican, et al., 2008), gender (e.g., Luh et al., 1991; Butler and Harvey, 2005, 2008), age and attractiveness (e.g., Burt and Perrett, 1997), and aesthetic preferences (e.g., Heath et al., 2005). Consistent with these findings, eye-tracking studies have found a preference for the left side of faces during the visual exploration of faces. For example, the participants' first gaze was more likely to be directed to the left side of a face, and the total fixation duration on the left side of a face was longer than that on the right side of the same face (e.g., Leonards and Scott-Samuel, 2005; Everdell et al., 2007; Hsiao et al., 2008; Guo et al., 2012; Samson et al., 2014).

In line with the above-mentioned behavioral findings, the left-side bias in face processing has also been shown in neuroimaging studies. For instance, the latency of the face-specific N170 event-related potential component is shorter when evoked by the left side of a face than the right side (Yovel et al., 2003). Studies using the functional magnetic resonance imaging (fMRI) technique have even linked the activation asymmetry of the fusiform face area (FFA) to the left-side bias in face recognition (Yovel et al., 2008). Together, both behavioral and neuroimaging studies have shown strong evidence for a left-side bias in face processing.

A similar left-side bias has also been observed for non-face stimuli, such as Chinese characters (e.g., Hsiao and Cottrell, 2009; Tso et al., 2014; Chung et al., 2017). Hsiao and Cottrell (2009) designed a perceptual judgment task that was similar to the chimeric face task but with mirror-symmetric Chinese characters as the stimuli. A clear left-side bias was observed in healthy adult Chinese readers. Tso et al. (2014) found that both expert writers and inexperienced writers similarly showed a stronger left-side bias effect that was uninfluenced by sensorimotor experience. The left-side bias effect disappeared when the stimuli were presented in an unfamiliar font (i.e., Feng). Furthermore, it has been shown that the left-side bias for Chinese characters was affected by a short-term task of reading directions and it was significantly reduced after a right-to-left reading task (Chung et al., 2017). Taken together, these findings indicate that the left-side bias is also reliably observed in Chinese character processing.

However, in the above-mentioned studies, the chimeric stimuli for choices were centrally presented and the reference face for the similarity judgment was presented either in the left or right visual field of the chimeric stimuli. Unfortunately, the studies did not focus on how the visual field of the reference face's presentation location affects the left-side bias. Previous studies, with the standard divided visual field paradigm, have shown that there is a left visual field (or right cerebral hemisphere) advantage for face recognition (Sergent and Bindra, 1981; Hillger and Koenig, 2007; Ramon and Rossion, 2012). Better performance has been observed for left visual field stimuli in several face-processing tasks, such as judging facial identity or emotional expression (e.g., Christman and Hackworth, 1993), the processing of upright/inverted faces (e.g., Ellis and Shepherd, 1975; Leehey et al., 1978), and composite face processing (e.g., Ramon and Rossion, 2012). The studies noted above suggested that the visual field affects the processing of faces in the divided visual field paradigm. However, the divided visual field paradigm had some differences from the chimeric paradigm of the left-side bias in face processing, wherein the reference faces were always presented in the left/right visual field with the chimeric face centrally presented simultaneously. It reminds us that the visual field of the reference stimulus may affect the left-side bias in the chimeric paradigm. We note, however, that in previous studies on the left-side bias in faces, the original faces were only presented in the left visual field (e.g., Brady et al., 2005). Although a recent study presented the original faces in both the left and right visual fields in half of the trials (e.g., Chung et al., 2017), the researchers did not investigate the influence of the visual field on the left-side bias. Therefore, it remains unclear whether in the chimeric paradigm, the presentation of the reference face in the left visual field has inflated the left-side bias in face processing.

Similar to face processing, the visual field of presentation also affects the processing of Chinese characters. Previous studies have shown a left visual field advantage in Chinese character processing across different paradigms (e.g., Tzeng et al., 1979; Cheng and Yang, 1989; Yang and Cheng, 1999); however, there is also evidence for a right visual field advantage (see Nguy et al., 1980). Especially, there is a left visual field advantage in orthographic processing. For example, when the orthographic similarity of two alternative items was manipulated, Yang and Cheng (1999) demonstrated a left visual field advantage in a character-matching task (see also Tzeng et al., 1979; Cheng and Yang, 1989). Taken together, these findings suggest that there is a left visual field advantage for the orthographic processing of Chinese characters (e.g., Tzeng et al., 1979; Cheng and Yang, 1989). However, it is unclear whether the visual field affects the left-side bias effect in Chinese character processing in the chimeric paradigm.

From the evidence discussed above, it has been shown that the left-side bias is stably observed for both faces and Chinese characters, and the advantage is observed when presenting the stimuli in the left visual field with the divided visual field paradigm. Unfortunately, how the visual field affects the left-side bias in face and Chinese character processing in the chimeric paradigm remains unclear. Therefore, in the present study, we investigated how the visual field of the reference stimulus' presentation modulates the left-side bias in face and Chinese character processing. The present studies used facial and no-face expert stimuli (e.g., Chinese characters). In Experiment 1, we examined the left-side bias in face recognition in healthy Chinese adults. In Experiment 2, we investigated whether the visual field of presentation of the original face would affect the left-side bias effect. In Experiment 3, we examined whether the influence of the presentation of the original stimuli of the left-side bias extends to the processing of non-face stimuli (i.e., Chinese characters). Based on the above-mentioned findings, we expected to observe reliable left-side biases for both face recognition and Chinese character processing. We also anticipated that a stronger left-side bias effect would be found when the original stimuli were presented in the left visual field for both faces and Chinese characters.

## Experiment 1: the left-side bias in face processing in Chinese adults

### Methods

#### Participants

Thirty-four healthy Chinese students (age range 18–21 years, mean 20 years, *SD* = 0.79, 22 females) were recruited from Zhejiang Normal University; they were paid for their participation. All participants only had the left-to-right reading habit. All participants reported that they were right-handed, and had normal or corrected-to-normal vision. Four participants were excluded from the analysis owing to their preference for pressing keys [e.g., the ratio for pressing the upper or lower keys was over 85%, which is beyond three standard deviations of the average of the left-side bias ratio (0.54 ± 0.09)]. The research protocols reported in Experiments 1, 2, and 3 were approved by the ethical committee of Zhejiang Normal University, and written informed consent was obtained from all participants (IRB Number: ZJUNPSY16023).

#### Stimuli

**Figure 1:**
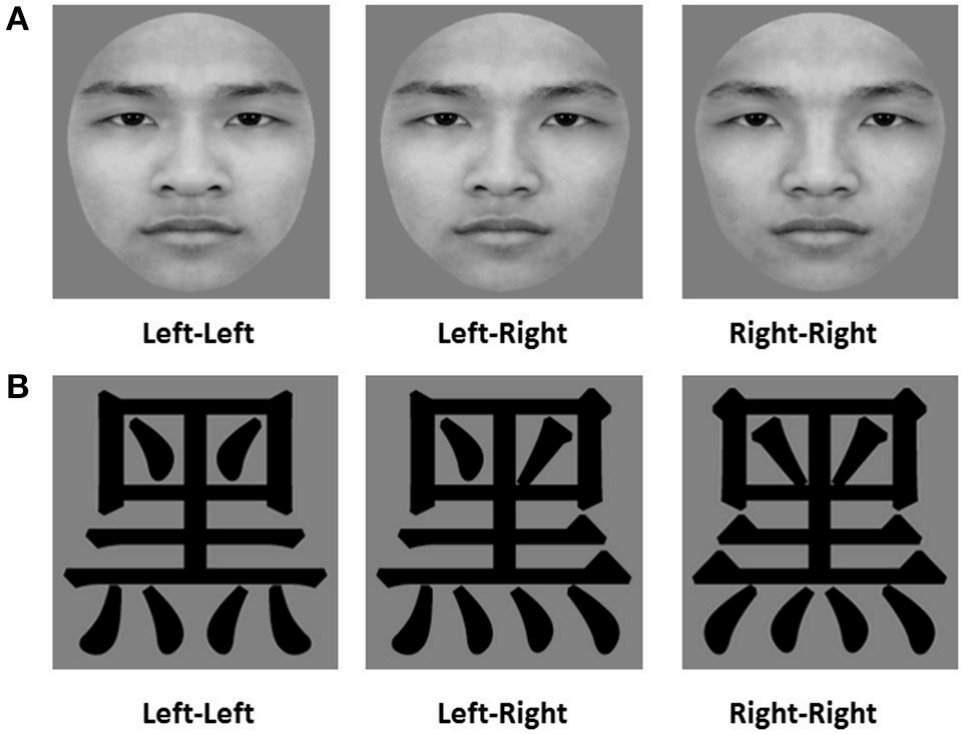
Examples of images for the chimeric face judgment task. **(A)** shows an example of the Chinese face stimuli, and **(B)** shows an example of the Chinese character stimuli. “Left-Left” denotes a left chimeric face/Chinese character image, “Right-Right” denotes a right chimeric face/Chinese character image, and “Left-Right” denotes an original face/Chinese character image.

Forty gray-scale pictures of Chinese faces (20 female faces) were selected from a set of faces used in previous work by our laboratory (Cao et al., 2015). All face images used in Experiment 1 displayed a neutral facial expression. All face images were cropped into a unified oval frame to remove the external features (e.g., hair, ears, and jawline). In order to investigate the left-side bias in face processing, we bisected each original face into two halves (left half and right half) along the vertical midline and combined each half face with its mirror image to create a new chimeric face. Thus, each original face made one left chimeric face and one right chimeric face. The final set of images included 40 original faces, 40 left chimeric Chinese faces, and 40 right chimeric Chinese faces (see Figure 1A). All of the stimuli subtended an angle of 6° × 7° from a viewing distance of 55 cm.

#### Procedure

**Figure 2:**
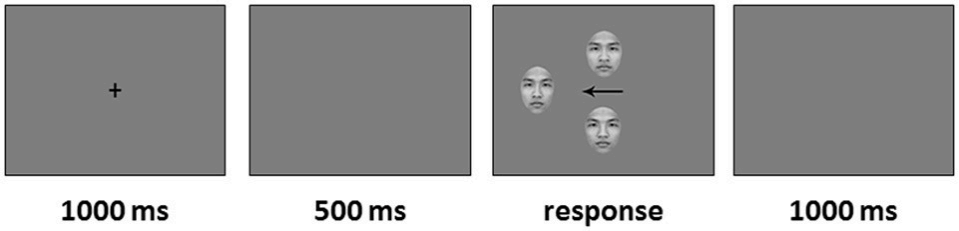
An example of the experimental procedure.

The participants sat on a chair in a dimly lit room, at a distance of 55 cm from a 17-inch CRT monitor (1,024 × 768 pixel resolution; 60 Hz refresh rate). All stimuli were presented against a light gray background. E-Prime 2.0 was used for stimulus presentation and behavioral response collection (Psychology Software Tools, Pittsburgh, PA). The chimeric face task contained 160 trials that were presented randomly in four blocks, and the block orders were random for each participant. The trials consisted of four types [the original face's location (left side, right side) × the left chimeric face's location (upper side, lower side)]. Each type contained 40 trials, and then each original stimulus was repeated four times. Each original face was presented on the screen simultaneously with its left and right chimeric face in each trial. In each trial, a fixation cross was first presented for 1,000 ms in the center of the screen, followed by a blank screen for 500 ms, and then the original face and its left and right chimeric images were presented on the screen simultaneously and remained until the participant's response. The original face was randomly presented on either the left side or the right side of the screen at a 7.5° visual angle away from the center. The two chimeric faces were presented randomly above and below a central arrow, with the arrow pointing to the original image. The edge-to-edge distance between the chimeric faces was about 6° (see Figure 2). After the response, there was a 1,000 ms inter-stimulus interval. Participants were asked to respond as quickly and accurately as possible by pressing the corresponding keys. The participants were asked to press “U” if they thought that the upper chimeric face was more similar to the original face than the lower chimeric face, and “V” if they thought that the lower chimeric face was more similar to the original face than the upper chimeric face.

#### Data analysis

The preference for the left chimeric face was calculated as the number of trials in which the participant chose the left chimeric face divided by the total number of trials. The response time was calculated from the onset of the presentation of the original face to the participant's response. The left-side bias refers to when the preference for the left chimeric face is significantly higher than the no-bias threshold (0.5).

### Results

#### Preference for the left chimeric face

**Figure 3:**
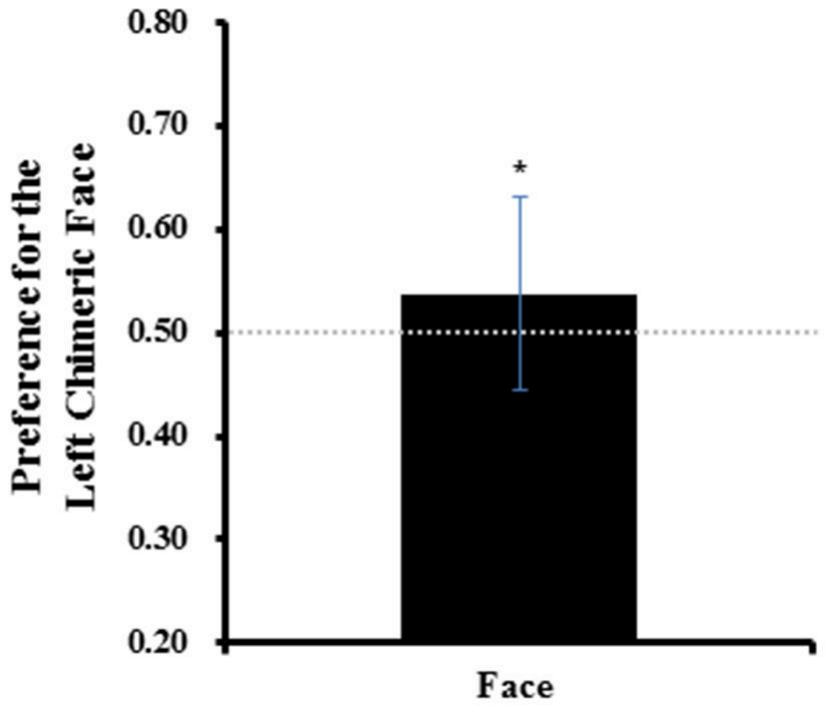
The preference for the left chimeric faces in Experiment 1. Error bars represent standard deviation of the means. ^*^p < 0.05.

One-sample *t*-tests comparing the average percentage of the left chimeric face being selected across all trials (*M* = 0.54 ± 0.09) to no-bias threshold (0.5) revealed a reliable left-side bias in Chinese face processing, *t*_(29)_ = 2.278, *p* = 0.030, Cohen's *d* = 0.42 (see Figure 3). The results suggested that a significant left-side bias effect appeared in Chinese face processing in Chinese adults.

#### Response time

A paired-samples *t*-test showed that there was no significant difference between the average response time for the left chimeric face selected across trials (*M* = 2,471 ± 796 ms) and the average response time for the right chimeric face selected across trials (*M* = 2,481 ± 790 ms), *t*_(29)_ = 0.255, *p* = 0.800. The results showed that there was no difference in response time between the participants selecting the left chimeric face or right chimeric face.

## Experiment 2: the role of the reference face's location for the left-side bias effect

Experiment 1 demonstrated that there is a stable left-side bias effect in face processing. However, it did not resolve how the visual field of the reference face location affects the left-side bias in face processing. Therefore, in Experiment 2, we investigated whether the visual field of presentation of the original face would affect the left-side bias.

### Methods

#### Participants

Thirty-one healthy university students (age range 17–23 years, mean 19.9 years, *SD* = 1.22, 15 males) participated in Experiment 2, who were in the habit of reading only left-to-right. None of them had participated in Experiment 1. One participant was excluded from the analysis because he did not finish the experimental task.

#### Stimuli

The stimuli used in Experiment 2 were the same as those in Experiment 1.

#### Procedure

The stimuli, task, and laboratory setup were the same as those in Experiment 1. To keep the trials in Experiment 2 in each visual field as the same number as in Experiment 1, the trials in Experiment 2 were twice as many in Experiment 1. In Experiment 2, a total of 320 trials were tested randomly in eight blocks, with the original face being presented in the left visual field in half of the trials, and in the right visual field in the other half of the trials. The block orders were random for each participant, and the presentation location of the reference face was randomly determined in each trial.

#### Data analysis

This experiment had a single-factor within-subjects design and the independent variable was the visual field of presentation of the original face (left vs. right visual field). The dependent variables of interest were the participant's preference for chimeric faces created from the left side of an original face, which was estimated in the same way as in Experiment 1, and the participant's response time.

### Results

#### Preference for the left chimeric face

**Figure 4:**
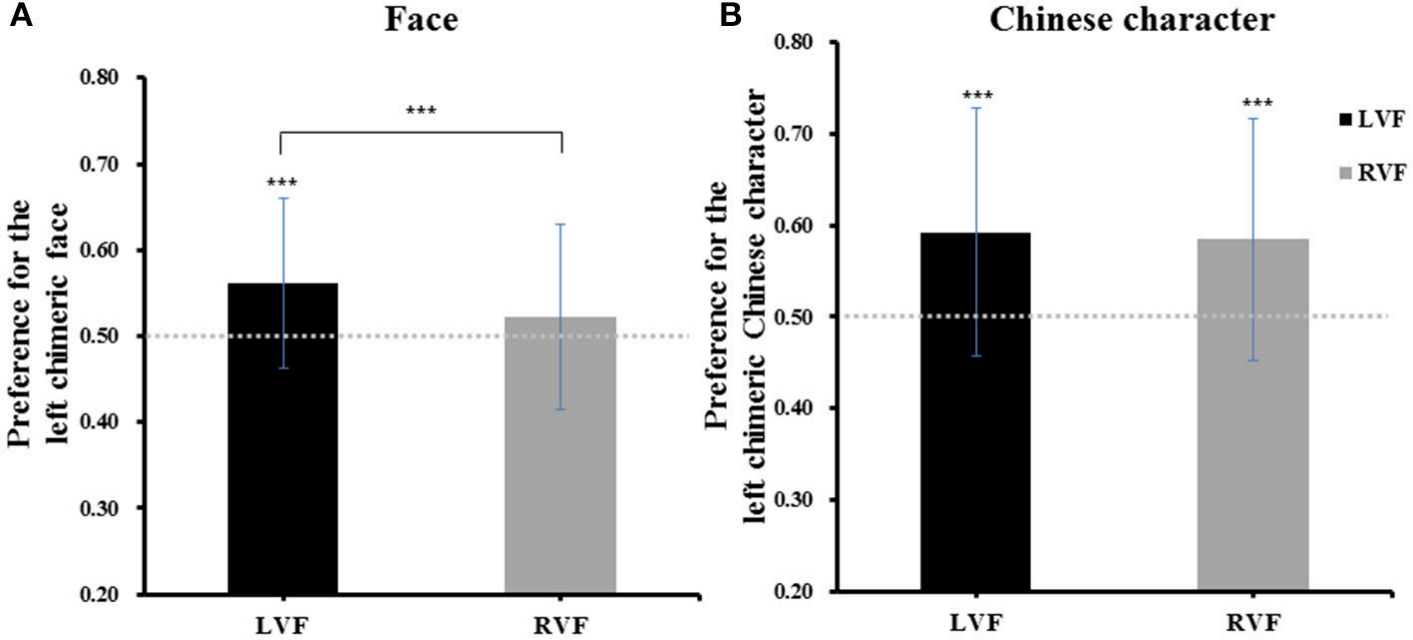
The results of Experiment 2 **(A)** and Experiment 3 **(B)**. “LVF” is the left visual field condition and “RVF” is the right visual field condition. Error bars represent standard deviation of the means. ^***^p < 0.001.

One-sample *t*-tests comparing the percentage of the left chimeric face being selected across all trials (*M* = 0.54 ± 0.10) to no-bias threshold (0.5) revealed a reliable left-side bias in face processing, *t*_(29)_ = 2.283, *p* = 0.030, Cohen's *d* = 0.42. The analysis also showed that the percentage of the left chimeric face being selected was higher for the left visual field condition (*M* = 0.56 ± 0.10) than for the right visual field condition (*M* = 0.52 ± 0.11), *t*_(29)_ = 4.177, *p* < 0.001, Cohen's *d* = 0.78. Importantly, the left-side bias was statistically reliable when the original face was presented in the left visual field condition, *t*_(29)_ = 3.435, *p* = 0.002, Cohen's *d* = 0.64, but not in the right visual field condition, *t*_(29)_ = 1.102, *p* = 0.280 (see Figure 4A). These results indicated that a left-side bias effect will appear only when the original face is presented in the left visual field.

#### Response time

A paired-samples *t*-test revealed that the response time in trials in which the left chimeric face was selected (*M* = 2,700 ± 1,060 ms) was roughly the same as that in trials in which the right chimeric face was selected (*M* = 2,739 ± 1,096 ms), *t*_(29)_ = 1.668, n.s. This observation was consistent with the finding of Experiment 1, which showed no difference in response times for selecting left and right chimeric faces. The analysis also showed that there was no response time difference for selecting the left chimeric face between the left (*M* = 2,687 ± 1,067 ms) and right (*M* = 2,716 ± 1,071 ms) visual field conditions, *t*_(29)_ = 0.648, n.s.

## Experiment 3: the role of the reference Chinese character's location for the left-side bias effect

### Methods

#### Participants

Thirty healthy university students (age range 18–24 years, mean 19.4 years, *SD* = 1.33, 18 females) participated in Experiment 3, who were in the habit of reading only left-to-right. None of them had participated in Experiment 1 or 2.

#### Stimuli

Forty mirror-symmetric Chinese characters were selected for use in Experiment 3. They were all high-frequency characters (Huang, 1994), with the number of strokes varying between 7 and 15. These characters were presented in a widely used standard typeface (Songti). As for the face stimuli in Experiments 1 and 2, the selected Chinese characters were bisected into left and right halves to create new chimeric Chinese characters. The left chimeric character was created from two left halves of the character and the right chimeric character was created from two right halves of the same character. A total of 40 original characters, 40 left chimeric characters, and 40 right chimeric characters were tested (see Figure 1B); all chimeric characters subtended 6° × 6° (visual angle) from a viewing distance of about 55 cm.

#### Procedure and design

The laboratory setup and task procedure were similar to that of Experiments 1 and 2, except that the participants were asked to make a speeded judgment about which chimeric Chinese character was more similar to the original character.

#### Data analysis

As in Experiments 1 and 2, the dependent variables were the participant's preference for the left chimeric character and their response times. The independent variable was the visual field in which the original Chinese character was presented (left vs. right visual field).

### Results

#### Preference for the left chimeric Chinese character

The results are plotted in Figure 4B. A one-sample *t*-test comparing the average percentage of the left chimeric character being selected across all trials (*M* = 0.59 ± 0.13) to chance level (0.5) revealed a reliable left-side bias in Chinese character processing, *t*_(29)_ = 3.728, *p* = 0.001, Cohen's *d* = 0.69. A paired-samples *t*-test showed that the average percentage of the left chimeric character being selected was not significantly different between the left visual field condition (*M* = 0.59 ± 0.13) and right visual field condition (*M* = 0.59 ± 0.13), *t*_(29)_ = 0.779, *p* = 0.442. The analysis also showed that the left-side bias was reliable in both the left visual field condition, *t*_(29)_ = 3.768, *p* = 0.001, Cohen's *d* = 0.70, and the right visual field condition, *t*_(29)_ = 3.527, *p* = 0.001, Cohen's *d* = 0.65. These results show that the left-side bias in Chinese character processing was not affected by the position of the original character.

#### Response time

A paired-samples *t*-test showed that there was no difference in response time between the selected left chimeric character (*M* = 1,629 ± 594 ms) and the selected right chimeric character (*M* = 1,663 ± 596 ms), *t*_(29)_ = 1.594, n.s. The response time for the selected left chimeric character was roughly the same in the left visual field condition (*M* = 1,620 ± 595 ms) and the right visual field condition (*M* = 1,637 ± 594 ms), *t*_(29)_ = 1.546, n.s.

## General discussion

The left-side bias is suggested to be a reliable behavioral characteristic of expert visual processing (Hsiao and Cottrell, 2009). Chimeric stimuli created from the left side of an overlearned stimulus are judged as being more similar to the original stimuli (e.g., Brady et al., 2005; Coolican, et al., 2008; Hsiao and Cottrell, 2009; Chung et al., 2017). Previous studies have demonstrated that this bias is consistently observed in face and Chinese character processing (e.g., Luh et al., 1991; Burt and Perrett, 1997; Hsiao and Cottrell, 2009; Balas and Moulson, 2011; Megreya and Havard, 2011; Tso et al., 2014; Proietti et al., 2015; Chung et al., 2017). The results of the present experiments confirm the left-side bias in face and Chinese character processing in Chinese adults. Our results and the results of previous studies together suggest that the left-side bias is a stable effect in expert visual processing.

The primary purpose of the present study was to examine how the visual field, where the original stimulus is presented in the chimeric paradigm, modulates the left-side bias in both face and Chinese character processing. In Experiments 1 (see the results of visual field analysis in the Appendix) and 2, the results showed that, for the first time, when an original face was presented in the left visual field, the preference for the left chimeric face was higher than no-bias threshold, but this effect was not observed in the right visual field. Supporting our speculation, these findings suggest that the visual field of presentation of the original face does affect the left-side bias in face processing. Many previous studies using a divided visual field paradigm have also repeatedly reported that the visual field modulates multiple aspects of face processing, as demonstrated in the inversion effect (e.g., Ellis and Shepherd, 1975; Leehey et al., 1978) and the composite effect (e.g., Ramon and Rossion, 2012). For instance, a stronger inversion effect was observed for faces presented in the left visual field than in the right visual field (e.g., Ellis and Shepherd, 1975; Leehey et al., 1978). In a recent study by Ramon and Rossion (2012), in which the authors investigated the lateralization of holistic processing using a divided visual field paradigm, a significant composite effect was observed in the left visual field, but not in the right visual field. Our finding and those from previous work using divided visual field paradigm suggest that the left visual field advantage may be a general property of the expert processing of faces across different paradigms.

Regarding the processing of Chinese characters, the results of Experiment 3 revealed a left-side bias in Chinese adults. This finding is consistent with previous work (e.g., Hsiao and Cottrell, 2009; Liu et al., 2013; Tso et al., 2014; Chung et al., 2017). Interestingly, following careful analysis of Hsiao and colleagues' studies, we found that experts who have learned traditional Chinese characters have a stable left-side bias effect. Additionally, both our results and Liu et al. (2013) showed that experts who have learned simplified Chinese characters also have a stable left-side bias effect. The findings noted above suggest that the left-side bias in Chinese character processing is a stable effect for experts who have learned both traditional Chinese characters and simplified Chinese characters. The findings support the hypothesis that the left-side bias is a stable marker of general perceptual expertise (e.g., Hsiao and Cottrell, 2009). Moreover, while our results have demonstrated that the left-side bias is equivalent in both the left and right visual fields in the chimeric paradigm, it is still unclear whether there is an equivalent visual field effect of left-side bias in the divided visual field paradigm. A recent study used the divided visual field paradigm found that experts learned traditional Chinese characters showed no composite effect in either the left/right visual field (Chung et al., 2015). Future studies should be designed to directly test the visual field advantage using the divided visual field paradigm combining with eye fixation measurement in Chinese experts learned simplified Chinese characters for left-side bias effect, inversion effect and composite effect.

Regarding our results, there was a left-side bias effect only when the original face was presented in the left visual field in the face processing, whereas for Chinese character processing, the left-side bias effect was significant in both the left and right visual fields. Many factors can account for the differential reference stimulus visual field advantage of the left-side bias in face and Chinese character processing. The most important factors may be the functioning, jointly or separately, of a specific perceptual processing area [FFA/visual word form area (VWFA)] and reading habits. Regarding the left-side bias effect in face processing, when the face was presented in the left visual field, it may be directly reflected in the right hemisphere (in the FFA), causing a significant left-side bias effect. Many previous studies have attributed the left-side bias effect in face processing to the right hemisphere dominance for face recognition (e.g., Gilbert and Bakan, 1973; Rhodes, 1985; Brady et al., 2005; Yovel et al., 2008; Proietti et al., 2015). In the meantime, the left-to-right reading habit may also affect the left-side bias in face processing. Many previous studies have found that the left-side bias effect was affected by the reading habit (e.g., Vaid and Singh, 1989; Sakhuja et al., 1996; Eviatar, 1997; Megreya and Havard, 2011). Therefore, when the original face is presented in the left visual field, the stronger left-side bias may be attributable to the collaboration of the specific perceptual processing area (FFA) and the left-to-right reading habit. However, when the original face is presented in the right visual field, both factors cannot contribute normally to the left-side bias processing, which leads to the absence of the left-side bias effect in the right visual field. For Chinese character processing, the specific perceptual processing area (VWFA) is in the left hemisphere for both, alphabetic languages and Chinese (e.g., Puce et al., 1996; Cohen et al., 2000; Liu et al., 2008; Kao et al., 2009; for reviews, see Dehaene and Cohen, 2011; Price and Devlin, 2011), which leads to more efficient processing of the Chinese character in the right visual field. Additionally, the left-to-right reading habit affects the left-side bias in Chinese character processing (Chung et al., 2017). Previous ERP/MEG studies demonstrated that the P100/M100 response of Chinese character recognition has generally exhibited a more right-lateralized activation in the visual system (Hsiao et al., 2007; Hsu et al., 2011; Wang et al., 2011; Sar, 2014). This right-lateralized advantage is attributed to the square shape and visual complexity of Chinese characters, which demands elaborate visual/spatial analyses in the right hemisphere (Tan et al., 2001). Therefore, when the original Chinese character is presented in the left visual field with the chimeric stimuli centrally presented simultaneously, the reading habit may facilitate reflection of the stimuli in the left visual field to the right hemisphere. Accordingly, facilitations due to the reading habit may play an important role in the left-side bias effect in Chinese character processing; however, when the original Chinese character is presented in the right visual field, the specific perceptual processing area (VWFA) may play an important role in the left-side bias effect. Due to the chimeric paradigm having some differences from the divided visual field paradigm, the results in the present studies could not be directly compared with results of the divided visual field studies. Of course, future research should directly test this hypothesis through behavioral and neurological studies across different paradigms.

Another factor that may account for the inconsistent visual field advantage between face and Chinese character processing may be the familiarity of the stimuli. While the Chinese characters were familiar to the participants in Experiment 3, and the participants were aware of the phonetic and semantic information of each Chinese character, the face images were unfamiliar to the participants in Experiment 2. Previous studies have found that the familiarity of the stimuli affected the left-side bias in face and Chinese character processing. For example, for the left-side bias effect in face processing, Brady et al. (2005) found that there were significant left-side bias effects for both familiar and unfamiliar faces, and the left-side bias effect was stronger for familiar faces than for unfamiliar faces. Regarding the left-side bias effect in Chinese character processing, a significant left-side bias has been found only for familiar Chinese fonts, and not for unfamiliar Chinese fonts (Tso et al., 2014). In our experiments, it was a limitation that we did not use different levels of the familiarity of faces and Chinese characters to directly explore the influence of familiarity on the left-side bias effect in the processing of faces and Chinese characters; therefore, future studies should examine whether the familiarity of expert stimuli affects the visual field advantage for the left-side bias effect.

In conclusion, consistent with previous studies, the present study found a significant left-side bias effect in the processing of both faces and Chinese characters. Interestingly, for face processing, the effect occurred when the original face was presented in the left visual field but not in the right visual field. For the processing of Chinese characters, the left-side bias was observed in both the left visual field and right visual field conditions. These results indicate a different visual field advantage for the left-side bias effect in the processing of faces and Chinese characters in the chimeric paradigm. The findings suggest that there are different characteristics underlying the left-side bias effect in the processing of faces and Chinese characters, and they imply that there are differences between the perceptual expert processing of faces and Chinese characters.

## Author contributions

CL and XC designed the experiments, performed the data analysis, and wrote the manuscript. CL executed the project.

### Conflict of interest statement

The authors declare that the research was conducted in the absence of any commercial or financial relationships that could be construed as a potential conflict of interest.

## Appendix

Analysis of the visual field in Experiment 1: The analysis showed that the percentage of the left chimeric face being selected was marginally higher for the left visual field condition (*M* = 0.55 ± 0.09) than for the right visual field condition (*M* = 0.52 ± 0.11), *t*_(29)_ = 1.871, *p* = 0.072, Cohen's *d* = 0.28. Importantly, the left-side bias was statistically reliable in the left visual field condition, *t*_(29)_ = 3.169, *p* = 0.004, Cohen's *d* = 0.42, but not in the right visual field condition, *t*_(29)_ = 1.183, *p* = 0.247.

The analysis for response time showed that there was no difference in selecting the left chimeric face between the left (*M* = 2,469 ± 818 ms) and right (*M* = 2,472 ± 798 ms) visual field conditions, *t*_(29)_ = 0.060, n.s.
